# Association between tennis training experience and executive function in children aged 8–12

**DOI:** 10.3389/fnhum.2022.924809

**Published:** 2022-08-03

**Authors:** Yue Xu, Wanxia Zhang, Hanfeng Zhang, Lijuan Wang, Yanlin Luo, Guoxin Ni

**Affiliations:** ^1^School of Sports Medicine and Rehabilitation, Beijing Sport University, Beijing, China; ^2^Sports Education Department, Beijing Jiaotong University, Beijing, China; ^3^Department of Neurobiology, Capital Medical University, Beijing, China

**Keywords:** tennis, physical activity, training experience, executive function, children

## Abstract

Cognitively engaging activities have been shown to facilitate the improvement of executive functions in children. However, a limited number of studies have investigated whether the relationship between dose parameters of physical activities and executive functions, and heterogeneity exists. In the present study, we aim to explore the association between tennis training experience and executive functions in children. Sixty children between the ages of 8 and 12 were recruited in this study and were allocated to the short-term (ST) group (<12 months, *n* = 30) and the long-term (LT) group (more than 12 months, n = 30). The abilities of inhibitory control, cognitive flexibility, and working memory were measured by the Stop-signal task, Switching task, and N-back task, respectively. There was no significant group difference in either the accuracy or reaction time of the Stop-signal task. No significant difference between the groups' accuracy in the Switching task was observed. However, the LT group presented a shorter reaction time than the ST group (731.69 ± 149.23 ms vs. 857.15 ± 157.99 ms, *P* < 0.01) in the Switching task. Additionally, training experience was positively associated with the reaction time of the Switching task. As for the N-back task, in comparison with the LT group, the ST group showed a longer reaction time (711.37 ± 168.14 ms vs. 164.75 ± 635.88 ms, *P* < 0.05). Moreover, training experience was also positively associated with the reaction time of the N-back task. But there was no significant group difference in the accuracy of the N-back task. In conclusion, children trained for over 1 year have better performance in cognitive flexibility and working memory than those trained in <1 year; thus, tennis experience is positively associated with executive functions.

## Introduction

As an important component of cognitive functions and behaviors, executive functions (EFs) are easy to observe and are often used to measure the gain of the sports on brain and cognition. Executive function is an umbrella term referring to a higher-order cognitive process that is responsible for problem-solving, self-regulation, and goal-directed behavior control (Wickel, 2017; Morgan et al., 2019). EFs are critical in our life since it allows us to think before we act (Diamond and Ling, 2016). Three distinct but interrelated components constitute executive function: inhibitory control (the ability to inhibit, downregulate, or delay the dominant, automatic, or prepotent responses, or to stay focused instead of being interrupted by temptations or distractions), working memory (the ability to keep the information in mind and further process the information), and cognitive flexibility (the ability to shift attention adaptively among multiple tasks, rules, mindsets, and perspectives and to deal with a sudden, unexpected situation in real-time) (Michel et al., 2019; Willoughby et al., 2019). Well-developed executive function contributes to academic achievement (reading, mathematics, and science) (Rhodes et al., 2016; Gerst et al., 2017; Willoughby et al., 2019) and greater behavioral self-regulation (Morgan et al., 2019). In the school setting, students with EFs deficits often present with academic difficulties and behavioral problems (Otero et al., 2014).

Childhood is a highly sensitive and critical period for executive function development due to development of the key cortex, the prefrontal cortex and late maturation (Michel et al., 2018; Chen et al., 2020). The developmental curve of gray matter for the frontal lobe peaks at around age 12 (Giedd et al., 1999). Accordingly, the executive function undergoes a protracted development. During the period from 7 to 12, all sub-components of the executive function experience significant development, consistent with increased gray matter density in the brain (Bidzan-Bluma and Lipowska, 2018). During this time, the development of executive function is more sensitive to environmental and external stimuli, such as sports activity (Ludyga et al., 2016; Takacs and Kassai, 2019). As common sense, physical activity improves physiological indicators, namely, metabolic biomarkers, cardio-respiratory functions, bone health, and muscular strength, which promotes physical fitness, decrease the risk of metabolic syndrome and cardiovascular disease, and reduces psycho-social problems. Recent evidence showed that there was a positive association between regular physical activity and brain functions (Ludyga et al., 2016; Poitras et al., 2016; Chen et al., 2020). Therefore, physical activity may elicit greater benefits for children.

As surmised above, the effects of various types of chronic exercises on cognitive functions in children have been studied extensively. Cognitively engaging activities (e.g., football, basketball, etc.) have been shown to benefit executive functions in children undergoing developmental changes (Ludyga et al., 2016; de Greeff et al., 2018). Compared with physically demanding intervention alone (simple repetitive aerobic exercises), cognitively engaging activities are more efficacious in facilitating executive functions in children between the ages of 6 and 12 years (Schmidt et al., 2015; de Greeff et al., 2018).

Tennis is a technical and tactical racquet sport asking for a complex combination of physical components, namely, strength, power, speed, agility, aerobic and anaerobic capacities, and neuromuscular coordination (Zagatto et al., 2018). Although studies have demonstrated that 12-month high frequency (four times per week) tennis play resulted in a greater improvement in working memory than low frequency (once a week) play (Ishihara and Mizuno, 2018) and tennis experience were positively related to cognitive flexibility (Ishihara et al., 2017a). They only compared differences between the genders and measured only one type of difficulty in working memory, set-shifting task in cognitive flexibility, and interference control in inhibitory control. As heterogeneity exists across studies, more studies are needed to conclude the dose parameters of different physical activities to achieve optimal cognitive improvement (Erickson et al., 2019). The purpose of this cross-sectional study was to investigate whether training experience will influence the promoting effect of tennis training on three sub-components of EFs (i.e., inhibitory control, working memory, and cognitive flexibility). It is hypothesized that longer tennis training experience is associated with better improvement in EFs.

## Methods

### Participant

PASS 11.0 (NCSS, LLC, USA) was used to perform the sample size calculation. The sample size was calculated to be at least 10 in each group, with power was 0.9, alpha was 0.05, and a lost-to-follow-up rate of 10% (Ishihara et al., 2017b). Finally, the proposed sample size for our study was 30 in each group. In total, 60 healthy children from a tennis club were recruited to participate in this study. Those subjects were included only if they fulfilled the following criteria: (1) between the ages of 8 and 12, (2) right-handed, (3) with normal intelligence and hearing, (4) with a normal or corrected vision, (5) without any color blindness, serious somatic disease, or nervous system injury, (6) had never participated in any similar psychological test as this study before, and (7) did not participate in any physical activity moderate-intensity or above other than tennis training.

Tennis training lessons were technique-based, comprising of three parts: (1) warm-up, (2) tennis training or competition, and (3) cool-down. Participants were categorized into two different groups based on training experience, which was measured by training records: (1) short-term group (ST): participants' training experience of fewer than 12 months and (2) long-term group (LT): participants' training experience more than 12 months. The study was conducted following the standards of the Declaration of Helsinki and was approved by the Ethics Committee of the Beijing Sport University. Written informed consent was given to participants and their parents before participation.

### Procedure

The present investigation was performed from May 2019 to September 2019. All participants and their parents were informed of the experimental procedure before the test and signed an informed consent form. Information on demographics, namely, age, gender, height, weight, and training parameters (frequency and experience) were collected before the tests.

Three computers were used to perform cognitive tasks. Stimulus test programs were coded by E-Prime (Psychology Software Tools Inc., Pittsburgh, United States). To avoid experimental errors caused by different physical sensations, all tests were conducted from 0:00 to 13:00 in a quiet room at a constant temperature of 23°C. Only one tester and one participant were allowed to be present in the room each time to avoid interference. Participants were asked to perform 10 trials identical to the formal test first. Formal tests were started when participants comprehend the experimental procedure and performed the task with no <90% accuracy (Wu et al., 2015). The experiments were conducted in the order of Stop-signal task, Switching task, and N-back task with appropriate rest between each test.

### Inhibitory control

The Stop-signal task was used to assess inhibitory control (Luo et al., 2021). A black prompt “+” was displayed for 500 ms in the center of the screen to remind participants to start the test. One of the four black geometrical figures (i.e., circle, triangle, rectangle, and diamond) was presented randomly soon afterward, sometimes followed by a STOP symbol with stimulus onset asynchronies (SOAs) of either 200, 400, 600, or 800 ms. Participants were instructed to press the left button of the mouse in the absence the of STOP symbol (Go trial), otherwise, inhibit the motor response (Stop trial). Each figure displayed for no more than 2,000 ms. If the reaction was too slow, the figure would disappear and the trial would be regarded as an error. There were 133 trials in total for each participant. Reaction time (RT, interval between stimuli and response) and accuracy (ACC, percentage of correct response) of the Go trial and the ACC of Stop trial were recorded in real-time.

### Working memory

The *N*-back task was used to assess working memory (Wolf et al., 2015). The task progressed in the order of difficulty level. One of the four black geometric figures (i.e., circle, triangle, rectangle, and diamond) was presented randomly on the screen. In this study, three blocks (the 0-back, 1-back, and 2-back tests) were administered. In the 0-back test, participants need to respond selectively according to the stimuli. Participants were instructed to press the left button of the mouse as soon as the triangle appears on the screen, or otherwise press the right button. In the 1-back test, participants were required to compare the current figure with the previous one and press the left button if the figures were identical, or otherwise press the right button. In the 2-back test, the subjects had to compare the current figure with the two figures presented previously and press the left button if they were identical, or otherwise press the right button. Each figure was presented for 500 ms, and the subjects had to respond within 2,000 ms. There were 46 trials in the 0-back test, 60 trials in the 1-back test, and 74 trials in the 2-back test altogether. RT and ACC of each participant were recorded in real-time.

### Cognitive flexibility

The Switching task was used to assess cognitive flexibility (Luo et al., 2021). The task began with the appearance of a prompt “+” in the center of the screen for 500 ms in either red or green color, followed by a pair of geometric figures (i.e., circle, triangle, rectangle, and diamond) placed horizontally in red or green, respectively. The figure with the same color as “+” was regarded as the target. The participants were asked to press the left button of the mouse if the target was the triangle, whereas the right button if not. If the participant was not able to respond within 4,000 ms, the figure would disappear and the trial would be considered an error. The participant would wait for another 500 ms and start the next trail. There were 96 trials in total for each participant, which were divided into three blocks. The first was the sustained condition, which contained two consecutive trails and two cues of the same color. The second was the switching condition, which contained two consecutive trails but the two cues were of different colors. The third was the sustained condition between the switching conditions, which had three or more consecutive trails and the cues are in two colors. RT and ACC of each participant were recorded in real-time.

### Data analysis and statistical analysis

SPSS 22.0 (IBM SPSS Statistics for Windows, IBM Corp., Armonk, NY) was used to perform data analysis. Normality was checked using the Shapiro–Wilk test. The demographic characteristics of the three groups were compared using the Mann–Whitney *U*-test. An analysis of covariance (ANCOVA) was used to control for confounding factors, such as age and gender. Two-way ANCOVAs were used to analyze the ACC of the Stop trial in the Stop-signal task [2 (group: ST, LT) × 4 (SOAs: 200, 400, 600, 800 ms)]. For the mean RT and ACC data obtained from the GO trail of the Stop-signal task, one-way ANCOVA was performed. The mean RT and accuracy data obtained during the other two EFs tasks were analyzed using 2 (group: ST, LT) × 3 (blocks) two-way ANCOVAs. Bonferroni *post-hoc* techniques were used if a significant main effect or interaction was discovered. The effect size in ANCOVAs was given as $\eta_{p}^{2}$ and was interpreted as follows: $\eta_{p}^{2}$ ≥ 0.01 = a small effect, $\eta_{p}^{2}$ ≥ 0.06 = a medium effect, and $\eta_{p}^{2}$ ≥ 0.14 = a large effect. Correlations between the mean RT or ACC data of the EFs tasks and groups or training experience were performed by calculating Pearson's correlation or Spearman's rank correlation coefficients (*r*). Correlation coefficient strength was classified as negligible <0.30, weak 0.31–0.50, moderate 0.51–0.70, or strong >0.71 (Sonesson et al., 2021). Statistical significance was set to *P* < 0.05 for all tests.

## Result

### Demographics

Participants' demographic characteristics are shown in Table 1. The demographic profile was found to be comparable, with no significant differences in gender, age, height, and weight between groups (*P* > 0.05). ST had significantly shorter training experience in comparison with LT (*Z* = −6.732, *P* < 0.001). As for training frequency, there was no significant difference between ST and LT.

**Table 1:** Demographic characteristics of the study participants.

**Variables**	**ST**	**LT**	***p*-value**
	**(*n* = 30)**	**(*n* = 30)**	**(*Z*)**
Male/female	19/11	18/12	0.792 (−0.263)
Age (years)	10.47 (0.82)	11.00 (1.14)	0.064 (−1.852)
Height (cm)	150.23 (5.64)	154.93 (10.93)	0.051 (−1.948)
Weight (kg)	39.67 (5.79)	41.90 (8.40)	0.287 (−1.065)
Training experience (month)	8.87 (4.33)^a^^***^	52.22 (17.07)	<0.001 (−6.732)
Training frequency (week)	2.00 (0.53)	2.40 (1.10)	0.203 (−1.273)

Values are presented as mean (SD). ST, the short-term group; LT, the long-term group.

*** statistically significant with P < 0.001.

a Compared with the long-term group.

### Inhibitory control

**Figure 1:**
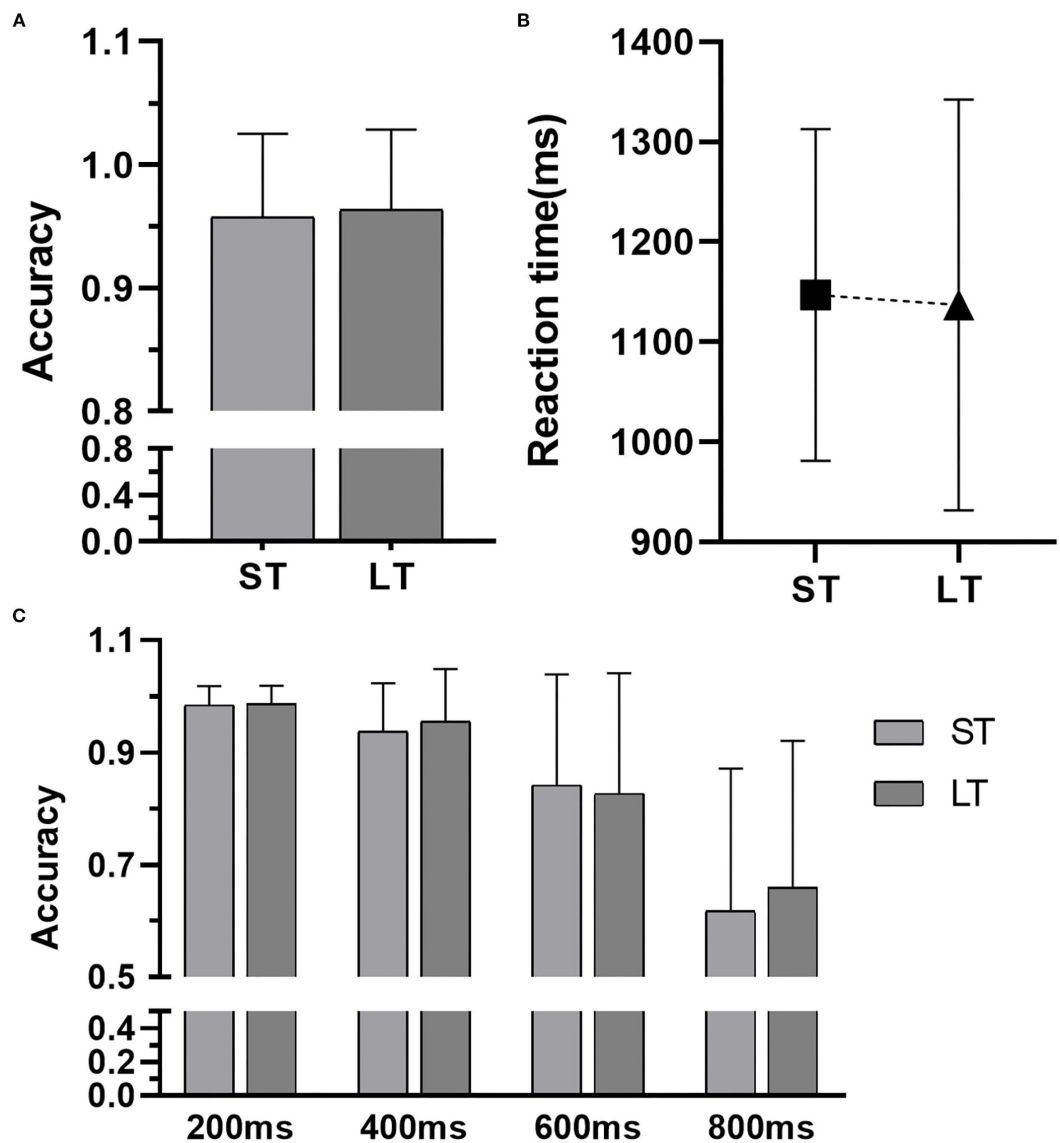
Results of the Stop-signal task. **(A)** The accuracy of the GO trails, **(B)** the reaction time of the Go trails, and **(C)** the accuracy of the Stop trails with different stimulus onset asynchronies. ST, the short-term group; LT, the long-term group.

For the GO trail of the Stop-signal task, there were no significant differences in the ACC and RT between groups (Table 2; Figures 1A,B), meanwhile, no significant correlation was apparent between the groups and the ACC and RT of GO trail (Table 2). For the Stop trial of the Stop-signal task, there were no Group × SOAs interaction effect [*F*_(3, 240)_ = 0.297, *P* = 0.828, $\eta_{p}^{2}$ = 0.004], or any group main effect [*F*_(1, 240)_ = 0.049, *P* = 0.825, $\eta_{p}^{2}$ < 0.001, Table 3; Figure 1C]. The significant main effect of SOAs was observed [*F*_(3, 240)_ = 49.315, *P* < 0.001, $\eta_{p}^{2}$ = 0.391], and ACC was significantly lower with the increase of SOAs (*P* < 0.001). No significant correlation was apparent between the groups and SOAs (Table 3).

**Table 2:** The accuracy and reaction time of the Go Trials in each group.

**Indicators**	**ST (*n* = 30)**	**LT (*n* = 30)**	***p-*value (*η^2^*)**	***F***	***p-*value (*r*)**
Accuracy (%)	95.77 (6.76)	96.35 (6.51)	0.769 (0.002)	0.087	0.422 (0.105)
Reation time (ms)	1,146.96 (165.81)	1,136.97 (205.37)	0.946 (0.000)	0.005	0.793 (−0.035)

Values are presented as mean (SD). ST, the short-term group; LT, the long-term group.

**Table 3:** The accuracy of the Stop trails at different interval times in each group.

**SOAs (ms)**	**ST**	**LT**	***p-*value**
	**(*n* = 30)**	**(*n* = 30)**	**(*r*)**
200	98.40 (3.41)	98.63 (3.26)	0.753 (0.042)
400	93.67 (8.64)	95.50 (9.35)	0.112 (0.207)
600	84.13 (19.75)	82.67(21.43)	0.927 (-0.012)
800	61.73 (25.41)	65.93 (26.15)	0.453 (0.099)

Values are presented as mean (SD). ST, the short-term group; LT, the long-term group; SOAs, stimulus onset asynchronies.

### Working memory

**Figure 2:**
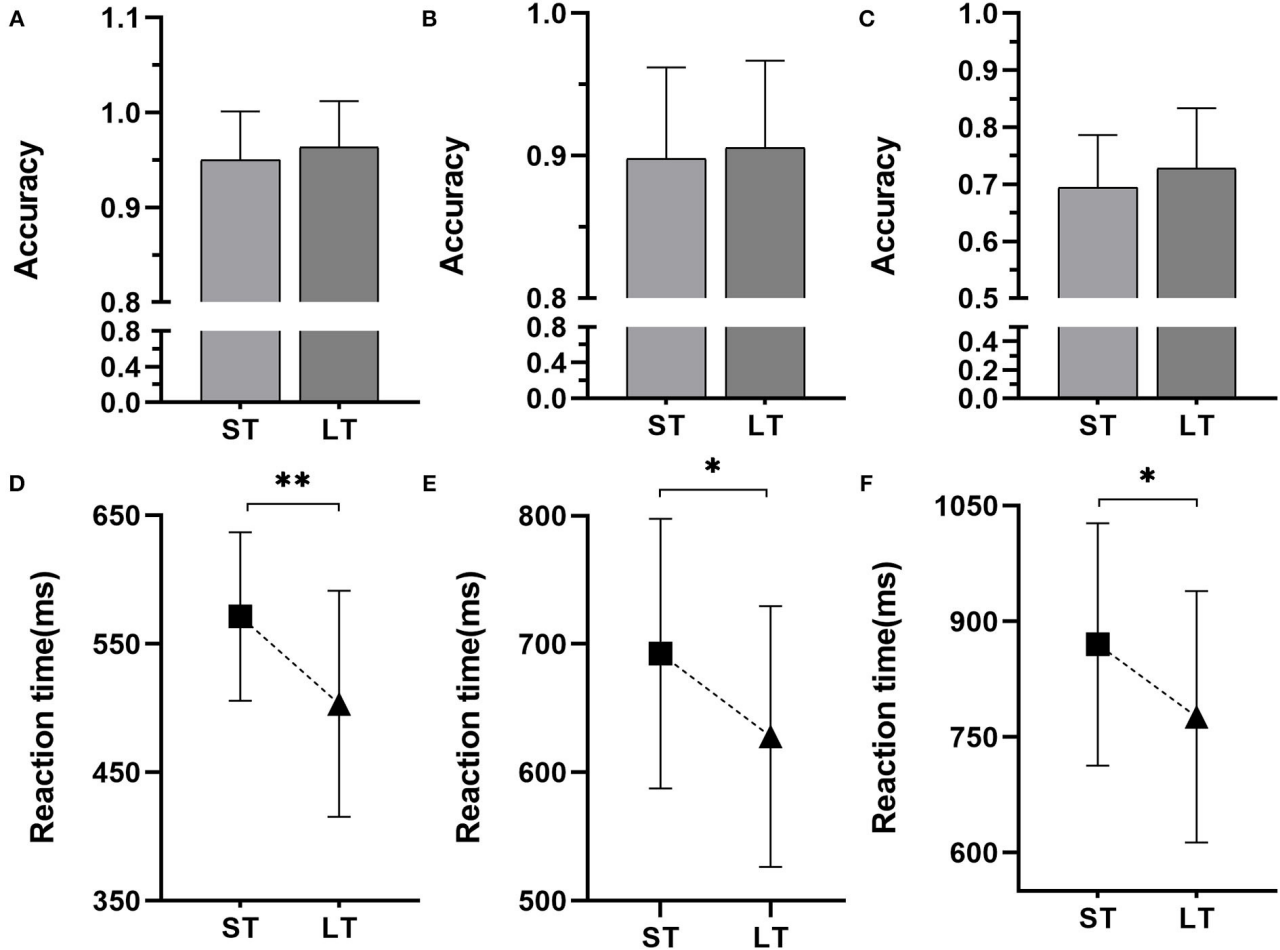
Results of the N-back task. **(A)** The accuracy of the 0-back test, **(B)** the accuracy of the 1-back test, **(C)** the accuracy of the 2-back test, **(D)** the reaction time of the 0-back test, **(E)** the reaction time of the 1-back test, and **(F)** the reaction time of the 2-back test. ST, the short-term group; LT, the long-term group. *Statistically significant with *P* < 0.05, **statistically significant with *P* < 0.01.

In the case of the RT, there was no significant interaction effect between group and block [*F*_(2, 180)_ = 0.287, *P* = 0.751, $\eta_{p}^{2}$ = 0.003] but there was a significant main effect of group [*F*_(1, 180)_ = 10.808, *P* = 0.001, $\eta_{p}^{2}$ = 0.059] and block [*F*_(2, 180)_ = 92.118, *P*< *0.001*, $\eta_{p}^{2}$ = 0.517]. An intergroup analysis showed that the RT of LT was significantly shorter than ST in the 0-back test (*P* = 0.001, Figure 2D), 1-back test (*P* = 0.015, Figure 2E), and 2-back test (*P* = 0.014, Table 4; Figure 2F). Furthermore, the RT increased with the increase of blocks (*P* < 0.001). In addition, a weak negative correlation between the groups and the RT of the 0-back test, 1-back test, and 2-back test (Table 4) was apparent. For the ACC of N-back task, there was no Group × Block (0-back test, 1-back, and 2-back test) interaction effect [*F*_(2, 180)_ = 0.540, *P* = 0.584, $\eta_{p}^{2}$ = 0.006] or group main effect [*F*_(1, 180)_ = 1.230, *P* = 0.269, $\eta_{p}^{2}$ = 0.007, Figures 2A–C]. Otherwise, there was a significant main effect of block [*F*_(2, 180)_ = 186.926, *P* < 0.001, $\eta_{p}^{2}$ = 0.785]. The ACC decreased with the increase of blocks (*P* < 0.001). No significant correlation was apparent between the groups and the ACC of the N-back task (Table 4).

**Table 4:** The accuracy and reaction time of the *N*-back task in each group.

**Block**	**Indicators**	**ST (*n* = 30)**	**LT (*n* = 30)**	***p-*value (*r*)**
0-back	Accuracy (%)	95.03 (5.09)	96.36 (4.83)	0.254 (0.150)
	Reaction time (ms)	571.34 (65.61)^a^^**^	503.33 (88.02)	0.001 (−0.431)^**^
1-back	Accuracy (%)	89.78 (6.43)	90.56 (6.13)	0.745 (0.043)
	Reaction time (ms)	692.59 (104.96)^a^^**^	628.01 (101.57)	0.013 (−0.318)^*^
2-back	Accuracy (%)	69.44 (9.19)	72.85 (10.48)	0.169 (0.180)
	Reaction time (ms)	870.18 (157.18)^a^^**^	776.30 (163.07)	0.013 (−0.320)*

Values are presented as mean (SD). ST, the short-term group; LT, the long-term group.

* Statistically significant with P < 0.05.

** Statistically significant with P < 0.01.

a Compared with the long-term group.

### Cognitive flexibility

**Figure 3:**
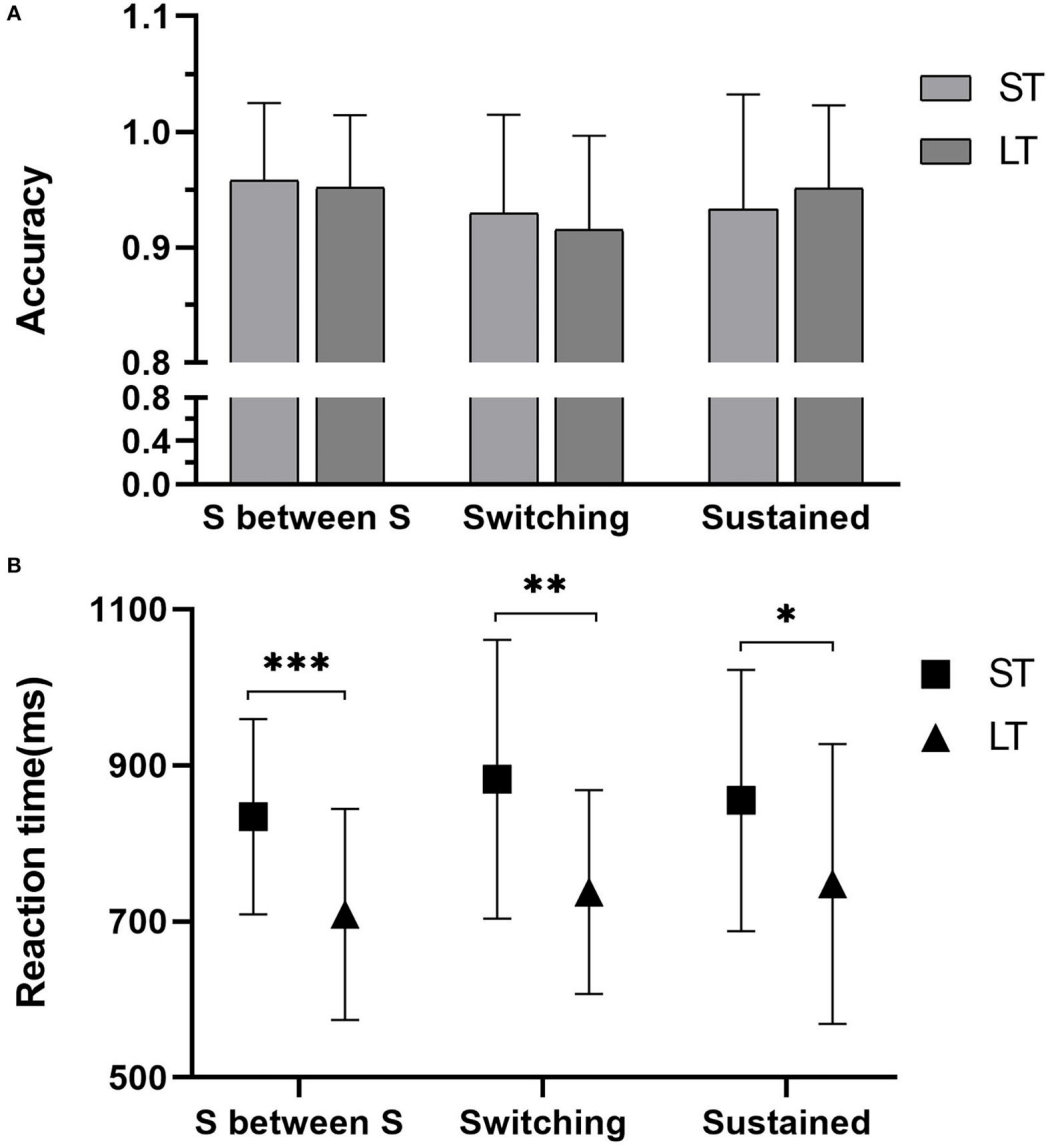
Results of the Switching task. **(A)** The accuracy of three blocks and **(B)** the reaction time of three blocks. ST, the short-term group; LT, the long-term group; S between S, Sustained between Switching block, *statistically significant with *P* < 0.05, **statistically significant with *P* < 0.01, ***statistically significant with *P* < 0.001.

Regarding the RT of Switching task, there were no Group × Block interaction effect [*F*_(2, 180)_ = 0.227, *P* = 0.797, $\eta_{p}^{2}$ = 0.003] or any block main effect [*F*_(2, 180)_ = 1.066, *P* = 0.347, $\eta_{p}^{2}$ = 0.012]. But significant group main effects were observed for RT measures [*F*_(1, 180)_ = 20.892, *P* < 0.001, $\eta_{p}^{2}$ = 0.108]. The RT of LT was significantly shorter than that of ST in the switching condition (*P* = 0.002), the sustained condition (*P* = 0.021), and the sustained condition between the switching conditions (*P* < 0.001, Table 5; Figure 3B). Meanwhile, there was a weak negative correlation between groups and the RT in the different conditions (Table 5). For the ACC of the Switching task (Figure 3A), there were no Group×Block interaction effect [*F*_(2, 180)_ = 0.749, *P* = 0.475, $\eta_{p}^{2}$ = 0.009] or any significant main effect of group [*F*_(1, 180)_ = 0.846, *P* = 0.359, $\eta_{p}^{2}$ = 0.005] and block [*F*_(2, 180)_ = 2.670, *P* = 0.072, $\eta_{p}^{2}$ = 0.030]. There was no correlation between the ACC of the Switching task and groups (Table 5).

**Table 5:** The accuracy and reaction time of the Switching task in each group.

**Block**	**Indicators**	**ST (*n* = 30)**	**LT (*n* = 30)**	***p-*value (*r*)**
Sustained between Switching	Accuracy (%)	95.79 (6.71)	95.14 (6.30)	0.546 (−0.079)
	Reaction time (ms)	834.22 (124.94)^a^^***^	709.12 (135.36)	0.000 (−0.454)^***^
Switching	Accuracy (%)	92.97 (8.50)^a^^**^	91.49 (8.17)	0.270 (−0.145)
	Reaction time (ms)	882.17 (178.43)^a^^**^	737.92 (130.51)	0.001 (−0.412)^**^
Sustained	Accuracy (%)	93.28 (9.93)	95.12 (7.16)	0.607 (0.068)
	Reaction time (ms)	855.07 (167.21)^a^^*^	748.02 (179.30)	0.020 (−0.300)^*^

Values are presented as mean (SD). ST, the short-term group; LT, the long-term group.

* Statistically significant with P < 0.05.

** Statistically significant with P < 0.01.

*** Statistically significant with P < 0.001.

a Compared with the long-term group.

### Correlation between training experience and EFs

**Figure 4:**
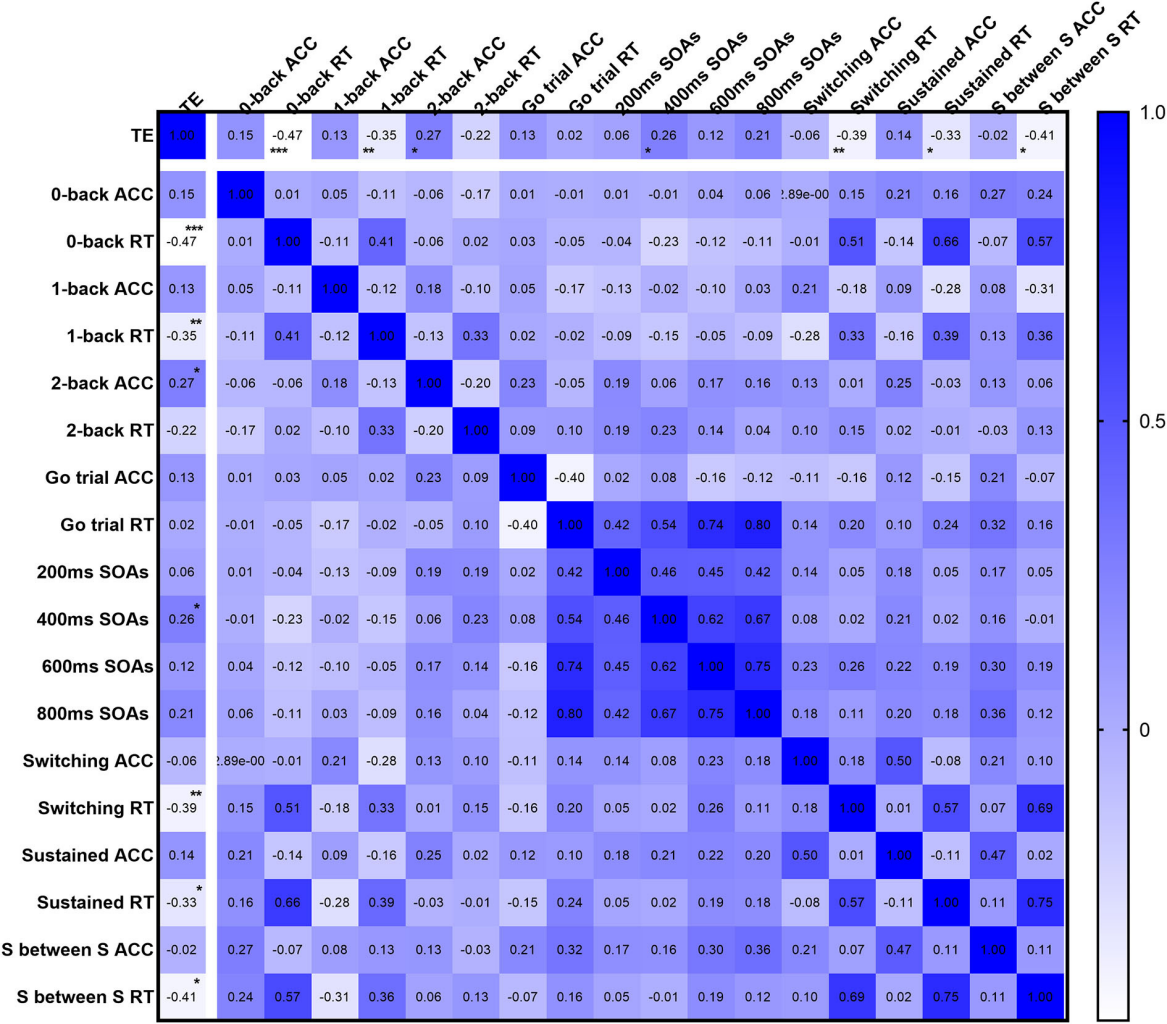
The heat map of the correlation coefficient (*r*) matrix. TE, training experience; ACC, the accuracy of each executive functions task; RT, the reaction time of each executive functions task; SOAs, stimulus onset asynchronies; S between S, Sustained between Switching block. *Statistically significant with *P* < 0.05, **statistically significant with *P* < 0.01, ***statistically significant with *P* < 0.001.

In terms of inhibitory control (Figure 4), the ACC in the 400 ms SOAs (*r* = 0.257, *P* = 0.047) was positively correlated with training experience, but the strength was negligible. As for working memory (Figure 4), there was a negligible positive correlation between the ACC of the 2-back test (*r* = 0.265, *P* = 0.04) and training experience. Meanwhile, there was a weak negative correlation between the RT in the 0-back test (*r* = −0.471, *P* < 0.001), 1-back test (*r* = –0.355, *P* = 0.005), and training experience. In the case of cognitive flexibility (Figure 4), a weak negative correlation between training experience and the RT in the switching condition (*r* = –0.391, *P* = 0.002), the sustained condition (*r* = –0.326, *P* = 0.011), and the sustained condition between the switching conditions (*r* = –0.413, *P* = 0.001) was observed.

## Discussion

In the present study, we examined the three domains of executive functions in children between the ages of 8 and 12 who participate in tennis training for more than 12 months and <12 months. We found that children between the ages of 8 and 12 with long-term training experience performed better than those with short-term training experience in cognitive flexibility and working memory. In addition, longer tennis training experience is associated with better performance in cognitive flexibility and working memory but not inhibitory control.

Our study found that longer training experiences were not associated with accuracy but with reaction time, regardless of the working memory loads. Children between the ages of 8 and 12 with long-term tennis training experience responded faster than children with short-term tennis training experience and had better performance in working memory. This may be since prolonged tennis training enhances the decision-making skills of the players and reduces their response delay times (Grigore et al., 2015). Previous studies have shown that people who regularly perform open-ended sports such as tennis and fencing exhibit faster reaction times, which is consistent with our study (Taddei et al., 2012; Šlosar et al., 2021). Working memory is the capacity to retain knowledge while making it accessible, and it is essential for processing all conscious information (Bergman and Söderqvist, 2017). Two reasons may explain why increased tennis training experiences improve children's working memory. First, working memory is likely to be activated during a child's engagement in tennis training because they must continuously recall tennis rules and techniques. Second, effective information processing, however, necessitates the use of working memory because children must maintain, update, and extract information that is pertinent to the task goal during tennis practice while ignoring or suppressing competing information that is irrelevant to the current context (Ishihara and Mizuno, 2018; Ishihara et al., 2018a,b). However, a recent study found that tennis training experience was not associated with working memory performance, which is inconsistent with our findings. The reason for this may be that they only used the difficult 2-back test, thus suggesting that future studies use the simple 1-back test to measure children's working memory (Ishihara et al., 2018b). Our study used three tasks with different memory loads to test the working memory performance of people with long- and short-term tennis training experience, yielding the same results as other studies that tested with only 2-back tests (Ishihara et al., 2017a). This study fills a gap in tennis research and further confirms the benefits of tennis training on working memory.

Another important finding was that increasing the training experience of tennis can enhance the cognitive flexibility of children. Additionally, longer tennis training experience is associated with better performance in cognitive flexibility tasks. Cognitive flexibility is the capacity to change one's attention and focus to pursue an internal objective or meet task demands (Garon et al., 2008). It is frequently assessed using a variety of task-switching and set-shifting tasks (Diamond, 2013). We obtained results consistent with previous studies without using a set-shifting task, reinforcing the benefits of increased tennis training experience to improve cognitive flexibility. The reason could be that children with longer tennis training experiences have more opportunities to switch between different types of tasks and problem-solving strategies, which are necessary to enhance cognitive flexibility (Ishihara et al., 2018b, 2019). Also, tennis places great demand on attention-shifting ability due to the need for tennis players to focus attention on the opponent's actions and balls, shift attention among different objectives, and make accurate and fast corresponding accordingly in a dynamically changing, unpredictable, and externally paced environment (Carlson et al., 1998; Shangguan and Che, 2018). During tennis play, for instance, players are required to memorize complex movement sequences, focus on the ball and opponent's position, as well as have attention-shifting capacities under time pressure. It is assumed that these activities activate the similar brain regions used to control the higher level of cognitive processes (e.g., cognitive flexibility) (Schmidt et al., 2015; Egger et al., 2019).

We were unable to determine the exact mechanisms behind the association between playing tennis and improved working memory and cognitive flexibility. Increased physical activity leads to physiological changes in the brain that might explain the current results. According to the studies, engaging in physical activity has a positive impact on brain structure and volume, namely, an increase in the white matter, parietal lobe gray matter, hippocampus, and basal ganglia volume (Chaddock et al., 2010; Niemann et al., 2014; Chaddock-Heyman et al., 2018). Additionally, physical activity is thought to affect brain neuroplasticity because it boosts the hippocampus's production of brain-derived neurotrophic factor (BDNF), encourages neuronal and synaptic growth and differentiation, and safeguards neuronal and synaptic transmission (Zhao et al., 2017). Moreover, recent research showed that exercise can increase blood flow to the brain, and clustering in exercise plasma reduced inflammation in the brain and enhances memory (De Miguel et al., 2021). Furthermore, another mechanism that may explain the improvement of cognitive performance might be that brain connectivity is enhanced following cognitive demanding exercise by increasing cell density and arborizing axons between brain structures engaged in motor and cognitive functions (Meijer et al., 2020).

However, we did not find a correlation between tennis training experience and inhibitory control. The correlation between tennis training experience and inhibitory control is not clear because (Ishihara et al., 2017c, 2018a) obtained different results using the same Stroop Color and Word task. Prepotent response inhibition and interference control are two commonly utilized distinctions in inhibitory control (Diamond, 2013). Tasks like the Go/no-go and Stop-signal are frequently used to test prepotent response inhibition, whereas the Flanker task and the Stroop task are commonly used to measure interference control (van der Bij et al., 2020). Unlike previous studies, in the present study, we used a Stop-signal task to explore the relationship between tennis training experience and prepotent response inhibition. However, we did not find a difference in prepotent response inhibition between the two groups. This phenomenon can be attributed to the fact that, unlike conflict tasks, the Stop-signal task does not require the execution of an alternative response and was not sensitive enough to detect any difference (Best and Miller, 2010). Moreover, because response inhibition grows better with age and our subjects were children between the ages of 8 and 12, the difficulty of the prepotent response inhibition task was insufficient to make a difference (Davidson et al., 2006). Ultimately, because the participants were all experienced in tennis training, the impact of tennis play on this population's inhibitory control may have had a ceiling effect (Ishihara and Mizuno, 2018).

In this study, we sought to assess differences in EFs in children with different tennis training experiences using a different type/difficulty of tasks than in previous studies and to explore the relationship between tennis training experience and three core components of EFs. However, several limitations should be acknowledged. Several potential moderators may influence executive function, namely, physical fitness level (Khan and Hillman, 2014; Borkertiene et al., 2019; Mora-Gonzalez et al., 2019), social-economic status (Vrantsidis et al., 2020), and peer and teacher–child relationship (van Lier and Deater-Deckard, 2016). Since we did not obtain this information, it is unknown if the aforementioned parameters account for variations in results. Given the cross-sectional nature of the study design, another limitation of the present study is that we were not able to infer a causal link between cognitively engaging physical activity and improvement of executive function. Therefore, longitudinal designs are needed in future research to make causal inferences. In addition, we only investigated whether training experience will influence the promoting effect of tennis training on three sub-components of EFs. Furthermore, since only behavior outcomes were assessed, the underlying mechanism of the promotion effect of cognitively engaging sports activities on executive functions was not obtained. Future studies should investigate brain biomarkers or structures to have a better understanding of biological or physiological pathways contributing to cognitive improvement. We only investigated whether training experience will influence the promoting effect of tennis training on three sub-components of EFs. In the future, it is indeed important to investigate whether closed-skill physical activity alone could affect EFs.

It is concluded that longer tennis experience was associated with better performance in working memory and cognitive flexibility in children between the ages of 8 and 12. Furthermore, tennis experience is positively associated with executive functions. The results of the present study may be of great practical importance for parents and educational settings to design physical activity programs that target the improvement of cognitive function for children.

## Data availability statement

The original contributions presented in the study are included in the article/supplementary material, further inquiries can be directed to the corresponding author/s.

## Ethics statement

The studies involving human participants were reviewed and approved by the Ethics Committee of Beijing Sport University (2019112H). Written informed consent to participate in this study was provided by the participants' legal guardian/next of kin.

## Author contributions

YX, WZ, and LW wrote the manuscript. HZ and YX analyzed the data. HZ recruited subjects and carried out the experiments to collect the data. YL conceived and planned the experiments. GN made critical revisions related to the important intellectual content of the manuscript. All authors contributed to the article and approved the submitted version.

## Funding

This study was supported by the Special Fund for Fundamental Scientific Research Expenses of Central Universities (20211003).

## Conflict of interest

The authors declare that the research was conducted in the absence of any commercial or financial relationships that could be construed as a potential conflict of interest.

## Publisher's note

All claims expressed in this article are solely those of the authors and do not necessarily represent those of their affiliated organizations, or those of the publisher, the editors and the reviewers. Any product that may be evaluated in this article, or claim that may be made by its manufacturer, is not guaranteed or endorsed by the publisher.
